# Modeling of High-Harmonic Generation in the C_60_ Fullerene Using Ab Initio, DFT-Based, and Semiempirical Methods

**DOI:** 10.1021/acs.jpca.3c07865

**Published:** 2024-03-27

**Authors:** Aleksander P. Woźniak, Robert Moszyński

**Affiliations:** Faculty of Chemistry, University of Warsaw, Pasteura 1, Warsaw 02-093, Poland

## Abstract

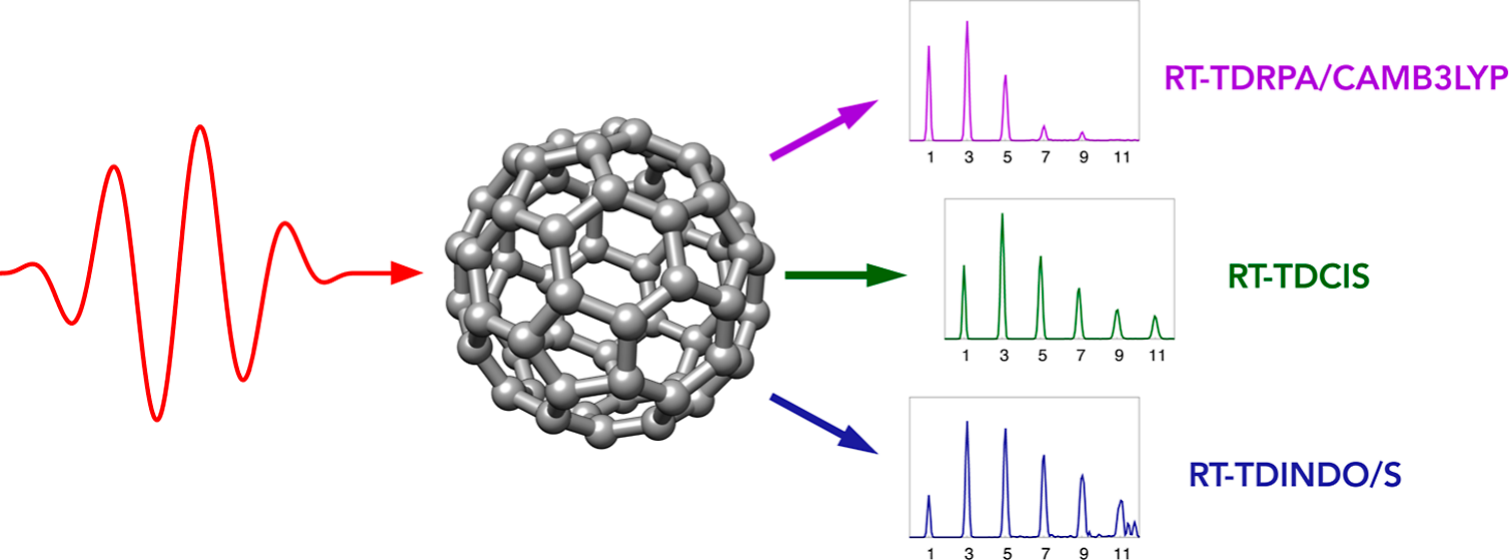

We report calculations of the high-harmonic generation spectra of the C_60_ fullerene molecule carried out by employing a diverse set of real-time time-dependent quantum chemical methods. All methodologies involve expanding the propagated electronic wave function in bases consisting of the ground and singly excited time-independent eigenstates obtained through the solution of the corresponding linear-response equations. We identify the correlation and exchange effect in the spectra by comparing the results from methods relying on the Hartree–Fock reference determinant with those obtained using approaches based on the density functional theory with different exchange–correlation functionals. The effect of the full random-phase approximation treatment of the excited electronic states is also analyzed and compared with the configuration interaction singles and the Tamm–Dancoff approximation. We also showcase the fact that the real-time extension of the semiempirical method INDO/S can be effectively applied for an approximate description of laser-driven dynamics in large systems.

## 1 Introduction

When atoms and molecules are subjected to extremely intense laser fields, their electron densities undergo rapid oscillations, emitting electromagnetic radiation that contains up to hundreds of harmonic frequencies of the incident light. The discovery of this phenomenon, known as high-harmonic generation (HHG), has ushered in the era of attophysics.^1,2^ It has enabled the routine production of ultrashort coherent electromagnetic pulses, making it possible to explore electron dynamics on previously unattainable time scales. Attosecond impulses have a wide range of applications, including, among others, molecular imaging,^3−5^ monitoring chemical and photochemical reactions in real time,^6,7^ determining molecular structures,^8,9^ studying photoionization,^10,11^ and investigating quantum coherence and electronic correlation effects.^12−15^

HHG was initially observed in noble^16−26^ and molecular^27−29^ gases, with the former remaining the most commonly used sources of harmonic radiation. In simple mono- or few-atomic systems, a single HHG event can be explained with the well-known three-step model (3SM),^30−34^ according to which an electron (1) is detached from the atom or molecule via tunneling ionization, (2) is accelerated away by the driving field and then reaccelerated back toward the parent ion when the field switches its sign, and (3) recombines with the parent ion, which leads to the emission of the radiation burst. HHG from gas targets, although relatively easy to achieve, is however hindered by a low conversion efficiency attributed to the low density of the medium.^35^ Thus, there is an ongoing search for novel and more efficient HHG sources. In recent years, thanks to the rapid development of experimental techniques, HHG has been demonstrated to also occur in bulk solids,^36−40^ liquids,^41^ and nanostructures,^42−47^ with the harmonic yield greatly exceeding that of atomic and molecular gases. The mechanism of HG in bulk media, although not fully revealed, is suspected to differ substantially from the 3SM.^36,38^

Progress in experimental discoveries in attophysics also necessitates the development of new theoretical methods capable of describing ultrafast electron dynamics in increasingly larger systems. In the past decade, there has been a significant rise in the popularity of employing quantum chemistry methods extended to the real-time domain for this purpose.^48−51^ These approaches are characterized by moderate computational costs typical of their time-independent counterparts, as well as a reasonable level of accuracy, allowing, for example, the consideration of multielectron effects. One of the most popular methods of this kind is the real-time time-dependent configuration interaction singles (RT-TDCIS), in which the time-dependent electronic wave function is represented as a linear combination of time-independent ground and singly excited electronic eigenstates of the examined system.^52−57^ Thanks to its highly favorable scaling with the number of electrons, it can be routinely applied not only to atoms^58−65^ and simple molecules^66−70^ but also to complex organic^71^ and biological^72−74^ compounds, often yielding results qualitatively or even quantitatively consistent with the experimental data.^71,72,75^ This raises the question of whether it can perform equally well for even larger systems such as nanostructured materials.

In this work, we report calculations of the HHG spectra of arguably the most well-known carbon nanostructure, the C_60_ fullerene molecule, carried out using quantum chemical approaches coupled to Gaussian basis sets. Fullerenes are currently of great interest in the field of attophysics as experimental studies report their exceptionally high HHG yield,^76−80^ significantly surpassing that of bulk carbon.^76,78,80^ This property is attributed to their high polarizability, as well as the occurrence of the plasmon resonance at the fullerene surface.^81^ From the theoretical point of view, HHG in fullerenes has been studied either using an extension of the three-step model^82,83^ or by using real-time simulations employing a variety of different approximate Hamiltonians. These include SAE-based models,^79,84^ tight-binding models,^85−87^ jellium-like approximation,^88^ and density functional theory (DFT) combined with pseudopotentials.^89,90^ However, for most of these models to perform properly, some form of system-specific parametrization is typically necessary, such as the construction of effective potentials. On the other hand, all-electron quantum chemical methods are much less system-dependent and offer a more universal simulation framework, which may prove very useful in possible future studies, such as investigating the role of various chemical modifications on HHG in nanostructures. Therefore, the first objective of this work is to assess whether the applicability of RT-TDCIS can be extended to large structures containing tens of atoms.

RT-TDCIS, being equivalent to the Hartree–Fock (HF) method for excited states, does not account for correlation effects. Although it has been demonstrated that dynamical electron correlation has little effect on the laser-driven dynamics in atoms^65,91−95^ and small molecules,^69,70,94,96,97^ in the case of C_60_, it is known that the single-determinant restricted HF wave function is not a stable ground state due to global correlations in the π orbital space.^98^ Therefore, limiting the calculations solely to RT-TDCIS may not be sufficient to obtain reliable HHG spectra. One prominent method for integrating dynamical correlation into the real-time quantum dynamics is the real-time time-dependent DFT (RT-TDDFT), in which the propagation is applied directly to Kohn–Sham spinorbitals forming the electron density.^99,100^ Similar to its time-independent variant, RT-TDDFT implicitly includes correlation effects through the chosen exchange–correlation potential. This approach is currently available in numerous Gaussian basis,^101−108^ plane wave,^109−112^ and grid-based implementations,^113−120^ among which the latter have gained the most popularity. Efficient algorithms for the propagation of the density matrix,^121,122^ often based on the Liouville–von Neumann equation,^103,114,123^ numerical techniques such as employing the Poisson equation to determine the Hartree potential,^116,117,124^ and commonly used adiabatic^125^ and local density^126^ approximations to the exchange–correlation potential greatly facilitate the solutions of both the time-independent and time-dependent DFT problems. Moreover, recent advancements in representing external electromagnetic fields, such as recasting Maxwell’s equations in the Schrödinger formalism,^114,127^ allow for effective and accurate description of light–matter interactions. Thanks to these advantages, grid-based RT-TDDFT finds widespread application in modeling linear and nonlinear optical responses in a broad spectrum of systems, from single atoms,^91,94,95,128^ through clusters^124,129,130^ and molecules,^50,94,96,131−133^ to nanostructures^134−137^ and solid-state materials.^115,127,138,139^ However, it also has its limitations. For instance, it incorrectly describes single-electron excitations and Rabi oscillations in closed-shell systems^140,141^ and suffers from the nonlinearity of the time-evolution equations.^142−144^ Additionally, its significant computational complexity typically restricts the treatment of larger systems at the all-electron level, necessitating the replacement of core electrons with pseudopotentials.^114,115,117,145^ Even with this workaround, the computational cost of RT-TDDFT greatly exceeds that of RT-TDCIS,^50^ a drawback shared with more sophisticated multideterminant methods such as real-time time-dependent coupled cluster,^93,146,147^ RT-TDCISD,^56,57,65,70,97^ and RT-TDCIS(D).^53,56,57^ An alternative approach to RT-TDDFT, proposed by Pauletti et al.^69^ and also utilized in this work, is to add the exchange–correlation potential directly to the RT-TDCIS Hamiltonian, effectively turning it into the real-time time-dependent counterpart of the Tamm–Dancoff approximation (TDA). Therefore, we compute the HHG spectra of the C_60_ molecule using RT-TDCIS and RT-TDTDA as well as their respective generalizations that include deexcitation terms: the real-time time-dependent extensions of the linear-response time-dependent Hartree–Fock method and the linear-response TDDFT (LR-TDDFT). This allows us to assess the role of multielectron effects in the laser-driven dynamics in fullerenes, which constitutes the second goal of this paper. Finally, we also calculate the HHG response using the semiempirical INDO/S Hamiltonian, which has been parameterized to reproduce excitation energies obtained with CIS in a limited active space.^148^ INDO/S has been recently extended to the real-time domain, but so far, it has only been employed in the modeling of absorption spectra in the perturbative regime, giving somewhat promising results.^149^ Thus, the third and final aim of this work is to investigate if INDO/S can also be applied for simulating strong field dynamics in large systems and used as a less expensive alternative to ab initio and DFT-based methods.

The paper is constructed as follows. In Section 2, we present a brief theoretical background of the used methods and provide computational details of the simulations. In Section 3, we present and discuss the results of the HHG calculations on the C_60_ fullerene. Finally, in Section 4, we conclude our work.

## 2 Methods

### 2.1 Theory

In this section, we provide an overview of the theoretical foundations for the methods used in the present work, collectively referred to as the real-time time-dependent single excitation-based methods. The common feature among all of them is providing an approximate solution to the time-dependent Schrödinger equation for the electronic wave function Ψ(*t*) within the Born–Oppenheimer approximation
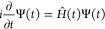
1where the time-dependent Hamiltonian *Ĥ*(t) consists of the time-independent ground-state molecular Hamiltonian *Ĥ*_0_ and the time-dependent interaction operator coupling the electrons to the laser field. In this work, the interaction operator is represented in the dipole approximation and in the length gauge
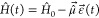
2where  is the molecular dipole operator and  is the external electric field. Since the ground state Hamiltonian  is Hermitian, its eigensolutions Ψ_*m*_ form a complete orthonormal basis set in the Hilbert space. Therefore, at any given point in time, the time-dependent wave function can be expanded as a linear combination of time-independent states Ψ_*m*_—among which we can distinguish the ground state Ψ_0_ and the excited states Ψ_*k*_—with time-dependent coefficients *C*_*m*_(*t*)
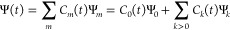
3Inserting eqs 2 and 3 into eq 1 leads to the equations for the time-evolution of the time-dependent coefficients
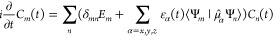
4where *E*_*m*_ is the eigenenergy of the *m*-th eigenstate and  is the α-th spatial component of the dipole operator. To obtain eq 4, we utilize the time-independent Schrödinger equation, , along with the orthonormality of the eigenstates, ⟨Ψ_*m*_|Ψ_*n*_⟩ = δ_*mn*_.

The eigenfunctions of  are known in an exact, analytic form only for the simplest model systems. However, for atoms and molecules containing more than one electron, they must be approximated by using some quantum chemical approaches. In all real-time time-dependent methods considered in this work, Ψ_0_ is assumed to be the ground-state closed-shell Slater determinant Φ_0_, built from real, orthonormal occupied molecular orbitals (MOs) ϕ_*i*_ represented in the linear combination of atomic orbital approximation
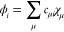
5In most electronic structure methods, Gaussian functions are chosen for the atomic orbital basis set χ_μ_ due to computational efficiency. The MOs ϕ_*i*_ are the solutions to the Hartree–Fock or Kohn–Sham self-consistent field (SCF) equations
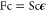
6where **F** is either the Fock or Kohn–Sham matrix, **S** is the overlap matrix, **c** is the matrix of coefficients *c*_μ*i*_, and **ϵ** is the diagonal matrix of MO energies. In addition to the occupied MOs, solving the SCF problem also provides a set of virtual MOs ϕ_*a*_.

The approximate excited states Ψ_*k*_ in eq 3 can be obtained by solving a linear-response equation specific to the particular real-time time-dependent method. The arguably most general linear-response theory considered in this work is the LR-TDDFT, based on the Kohn–Sham reference determinant. The (non-Hermitian) LR-TDDFT eigenequation reads^150^
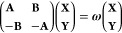
7Here, the matrices **A** and **B** are commonly referred to as the excitation matrix and the deexcitation matrix, respectively. Their elements are defined as
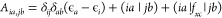
8
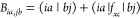
9where *i* and *j* denote occupied MOs (hole states) ϕ_*i*_ and ϕ_*j*_, *a* and *b* denote virtual MOs (particle states) ϕ_*a*_ and ϕ_*b*_, ϵ_*i*_ and ϵ_*a*_ are the occupied and virtual MO energies, respectively, and *f*_*xc*_ is the exchange–correlation kernel used in the SCF procedure. The two-electron Coulomb integrals (*ia*|*bj*) and exchange–correlation integrals (*ia*|*f*_*xc*_|*bj*) are written in the Mulliken notation. According to Casida,^151^ every excited state Ψ_*k*_ with the excitation energy ω_*k*_ = *E*_*k*_ – *E*_0_ is defined by the vectors **X**_*k*_ and **Y**_*k*_ as
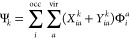
10where Φ_*i*_^*a*^ constitute the set of singly excited Slater determinants. The vectors **X**_*k*_ and **Y**_*l*_ also fulfill the normalization condition
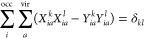
11

If the orbitals used to construct the matrices **A** and **B** are obtained from the Hartree–Fock method, the exchange–correlation kernel *f*_*xc*_ is replaced by the (nonlocal) HF exchange kernel *f*_HF_, (*ia*|*f*_*xc*_|*bj*) = (*ia*|*f*_HF_|*bj*) = −(*ib*|*aj*), and eq 7 is reduced to the linear-response time-dependent Hartree–Fock (LR-TDHF) method, also known as the random-phase approximation (RPA).^150^ It is worth mentioning that despite their similarities, LR-TDHF and LR-TDDFT have, in fact, been derived independently from each other, with the former being significantly older.^152,153^ However, in this work, it is more convenient for us to treat LR-TDHF as a special case of LR-TDDFT, since we aim to investigate the effect of the exchange–correlation potential on the laser-driven electron dynamics.

The terms LR-TDHF and LR-TDDFT have traditionally been used for the methods solely for obtaining the excited eigenspectra of atoms and molecules. On the other hand, RT-TDHF and RT-TDDFT usually refer to the single-determinant approaches in which the real-time propagation is applied to MOs. Moreover, some authors use the term RPA interchangeably not only with LR-TDHF but also with LR-TDDFT.^154−156^ In order to avoid possible confusion, we make use of this fact in this work and refer to the method of propagating the time-dependent wave function (3), expanded in the basis of states obtained through the solution of eq 7, as RT-TDRPA-xc, where xc may stand for HF, DFT, or any particular exchange–correlation functional.

Setting the deexcitation matrix **B** to zero in the LR-TDDFT (LR-TDHF) equation leads to the well-known TDA (CIS) approximation^150^
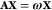
12The properties (10) and (11) also apply to TDA and CIS states, with the exception that **Y**_*k*_ = 0 for every Ψ_*k*_. TDA (CIS) approximation already provides a significant simplification of the linear-response problem compared to the full LR-TDDFT (LR-TDHF) as eq 12 is Hermitian. This allows for a reduction in the computational cost required to obtain excited states as well as helps avoid various numerical issues, with the most infamous being the triplet instabilities.^157^ However, TDA (CIS) still necessitates calculations of all of the one- and two-electron integrals required to solve the ground-state Kohn–Sham (Hartree–Fock) equations and to construct the excitation matrix **A**, which can be prohibitive for very large systems.

To overcome this bottleneck, various semiempirical quantum chemical methods have been historically proposed, all of which rely on setting certain types of integrals to zero or replacing them with much simpler parametrized formulas. Although the parameters of most semiempirical methods have been adjusted to reproduce the results of ab initio ground-state calculations,^158^ one of them, named INDO/S, has been specifically constructed to yield excitation energies matching those of CIS in a truncated active orbital space.^148,159^ The detailed description of INDO/S can be found elsewhere,^148,160−162^ while here we briefly discuss only the key assumptions of this method. Unlike in the ab initio and DFT-based methods, and similarly to other semiempirical methods, the MOs in INDO/S are expanded not in a Gaussian basis set but in the minimal basis set of Slater orbitals that describe only the valence orbitals of every element. INDO/S originates from the Hartree–Fock formalism, with the elements of the Fock matrix defined in the atomic orbital representation as
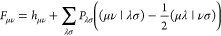
13where *h*_μν_ are the elements of the one-electron Hamiltonian matrix and *P*_λσ_ are the elements of the one-electron density matrix, . However, the solution of the SCF problem is heavily simplified due to the so-called zero differential overlap approximation, which sets the overlap matrix **S** in eq 6 to an identity matrix, *S*_μν_ = δ_μν_. As a consequence, all three- and four-center two-electron integrals between basis functions (μν|λσ) vanish. The two-center two-electron integrals are also set to be equal to zero, with the exception of Coulomb-like integrals involving only two basis functions, , which are calculated from the Mataga–Nishimoto formula^163^ (the superscripts *A* and *B* denote different atomic centers). The one-center two-electron integrals are estimated by using the Slater–Condon Coulomb and exchange factors. The one-electron integrals *h*_μν_ are also approximated using combinations of one-electron core integrals, resonance integrals, modified overlap integrals, and two-center two-electron integrals.^148^ All approximations used during the construction of the semiempirical Fock matrix are also applied when constructing the INDO/S excitation matrix **A**.

Having obtained the set of electronic states Ψ_*k*_ using the linear-response theory of choice and before proceeding to propagate the time-dependent wave function Ψ(*t*), we also need to determine the elements of the dipole moment operator in eq 4. Their values, particularly the dipole moment expectation values of the excited states ⟨Ψ_*k*_|μ_α_Ψ_*k*_⟩ and the dipole transition moments between two excited states ⟨Ψ_*k*_|μ_α_Ψ_*l*_⟩, can be determined with high accuracy using quadratic response theory.^164^ However, since we aim to propagate the time-dependent wave function using the full eigenspectrum of eq 7, which for systems as large as C_60_ may consist of tens of thousands of states, this approach would be practically unfeasible. Therefore, we calculate the dipole operator elements in an approximate manner by inserting eq 10 directly into ⟨Ψ_*m*_|μ_α_Ψ_*n*_⟩^165^
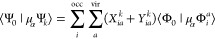
14
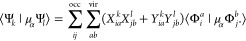
15The dipole moment integrals between the ground and excited Slater determinants can be readily evaluated using the Slater–Condon rules. For CIS, TDA, and INDO/S states, these expressions are reduced to
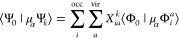
16
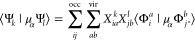
17

To solve eq 4, we introduce time discretization and propagate the wave function using the second-order split-operator technique
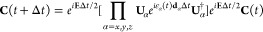
18where Δ*t* denotes the time step, **E** is the diagonal matrix of  eigenenergies, and the unitary matrix **U**_α_ diagonalizes the α-th dipole component matrix **μ**_α_, **U**_α_^†^**μ**_α_**U**_α_ = **d**_α_.

Similarly to our previous works^64,65,70,75^ and to the works of other authors utilizing real-time time-dependent methods to simulate strong-field electron dynamics,^58−60,62,68,69,71−74^ we employ the heuristic finite lifetime model of Klinkusch et al.^166^ to compensate for the incompleteness of the atomic orbital basis sets. The electronic energies *E*_*k*_ of excited states beyond the ionization threshold are modified by adding imaginary ionization rates
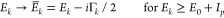
19The finite lifetime model was originally developed for RT-TDCIS^166^ and later extended to RT-TDCI with higher excitations.^65,167^ The ionization rates of CIS states are defined as
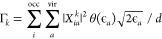
20where θ(*x*) is the Heaviside step function and the empirical parameter *d* represents a maximum distance from the molecule that a (semiclassical) electron can travel before undergoing ionization. Naturally, eq 20 applies to the TDA and INDO/S states as well. In this work, we also extend the finite lifetime model to RT-TDRPA. By analogy with eq 20, we define the heuristic ionization rates of LR-TDDFT and LR-TDHF states as
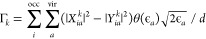
21The motivation behind eq 21 is as follows. The ionization rate of every CIS state (20) can be interpreted as a sum of excitation probabilities to individual virtual orbitals  multiplied by the ionization rates of these orbitals  (the Heaviside function ensures that only the virtual MOs with positive energies are ionizable). Since the RPA theory accounts for both excitations and deexcitations, the ionization rates of LR-TDHF and LR-TDDFT states have to be accordingly reduced by the probabilities  that an electron becomes deexcited from the virtual MO ϕ_*a*_ back to the occupied MO ϕ_*i*_.

Once the time-dependent wave function Ψ(*t*) is known, the HHG spectrum of the examined system *I*_HHG_ can be calculated from the Fourier transform of the optical response, which, in this work, is taken to be the dipole acceleration ,
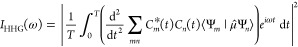
22where *T* is the total propagation time.

### 2.2 Computational Details

We performed calculations of the HHG spectra of the C_60_ molecule subjected to short, intense laser pulses at the RT-TDRPA-DFT, RT-TDRPA-HF, RT-TDTDA, RT-TDCIS, and RT-TDINDO/S levels of theory. The geometry of C_60_ was optimized at the B3LYP-D3/cc-pVTZ level of theory using Gaussian16 (Rev. C.01) software.^168^ The RT-TDRPA-DFT and RT-TDTDA calculations are performed in two variants, utilizing two different exchange–correlation functionals. The first one is the standard B3LYP hybrid functional,^169^ frequently employed in calculations involving fullerenes and their derivatives.^170−172^ The second one is its Coulomb-attenuated version, CAM-B3LYP,^173^ which is reported to perform better than B3LYP in describing excited states^174,175^—a feature that may be important for the correct description of HHG. Since we consider the time-evolution of a closed-shell system in the absence of spin-dependent perturbations, we are only interested in the singlet excited manifold, so the linear-response equations are constructed using singlet configuration state functions rather than pure Slater determinants. We obtain the **A** and **B** matrices and the dipole moment integrals used in the RT-TDRPA, RT-TDTDA, and RT-TDCIS calculations using a modified version of the PySCF 2.4 package.^176^ The semiempirical **A** matrices used in the RT-TDINDO/S calculations and the dipole moment integrals between INDO/S configurations are generated using a modified version of MOPAC22.^177−179^ The solution of the linear-response equation, the construction and diagonalization of the dipole moment matrices **μ**, and the real-time propagation are performed using a homemade program. All utilized approaches employ a full diagonalization of either eq 12 or eq 7. The LR-TDDFT and LR-TDHF equations are solved using the Cholesky decomposition technique.^180^

In the simulations, the external laser field is represented by a linearly polarized electric field pulse with a sine-squared envelope
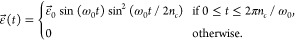
23The cycle-averaged laser intensity *I*_0_, related to the field amplitude  via , is set to 5 × 10^13^ W/cm^2^, and the number of optical cycles is *n*_*c*_ = 10. We use two values of carrier frequency ω_0_, 1.55 and 0.95 eV, corresponding to the wavelengths of 800 and 1300 nm, respectively. The 800 nm pulse has the duration of 1103 au (≈26.7 fs), while the 1300 nm pulse has the duration of 1793 au (≈43.4 fs). Due to the high symmetry of C_60_ and computational limitations, we consider only one polarization vector, parallel to one of the *S*_6_ improper axes. The wave functions are propagated using the time step Δ*t* = 0.01 au, a value that ensured the convergence of the obtained results. Every propagation starts from the single-determinant ground state, which serves as a reference for the corresponding real-time time-dependent method. Also, the value of *I*_*p*_ used in the finite lifetime model is estimated from the Koopmans’ theorem as the negative energy of the highest occupied MO—ϵ_HOMO_—within the reference state.

The HHG spectra are computed from the dipole acceleration obtained by numerically differentiating the time-resolved dipole moment twice with respect to time. We also apply the Hann window to the dipole acceleration before taking the Fourier transform in order to account for the finite simulation time.

## 3 Results and Discussion

### 3.1 Optimization of the HHG Simulation Framework

When performing quantum-chemical calculations in the strong-field nonperturbative regime, three factors have the most significant influence on the accuracy of reproducing laser-driven electron dynamics:

(a) the employed Gaussian basis set. Given that multiple transitions to highly excited and unbound electronic states are an inherent part of HHG, it is desired to simulate the electron dynamics using the most accurate available representation of the electronic continuum. It is worth noting that in our case, the choice of the basis set pertains only to the ab initio and DFT-based methods as INDO/S has been designed to work solely with the minimal Slater basis set;
(b) the size of the active orbital space. When the size of the simulated system becomes considerable, a full configurational space with excitations from all occupied MOs to all virtual MOS can no longer be employed in the calculations, especially if a large basis set is used and the propagation involves the entire eigenspectrum of the linear-response equation. In our earlier calculations on the H_2_ molecule, we demonstrated that truncating the virtual orbital space may be suboptimal because excluding the highest-lying virtual MOs may negatively affect the description of all excited states, not just those with the highest energies.^70^ At the same time, other studies indicate that HHG is usually dominated by transitions from several of the highest-lying occupied MOs,^74^ so excluding excitations from core orbitals may be a preferred option for reducing the number of configurations; and
(c) the parametrization of the applied wave function absorber. In the finite lifetime model employed in our calculations, the absorption rate is governed by the escape length value *d*. Ideally, the absorber should selectively eliminate components of the wave function that cannot be accurately represented by the basis set without interfering with the HHG process. In practical applications involving atoms and moderately sized molecules, known to generate harmonics in accordance with the three-step model, a value of *d* close to the maximum electron excursion distance in the laser field, *E*_0_/ω_0_^2^, is usually selected. However, since HHG in nanostructures is known to deviate from the 3SM, the proper choice of *d* requires additional investigation.

**Figure 1:**
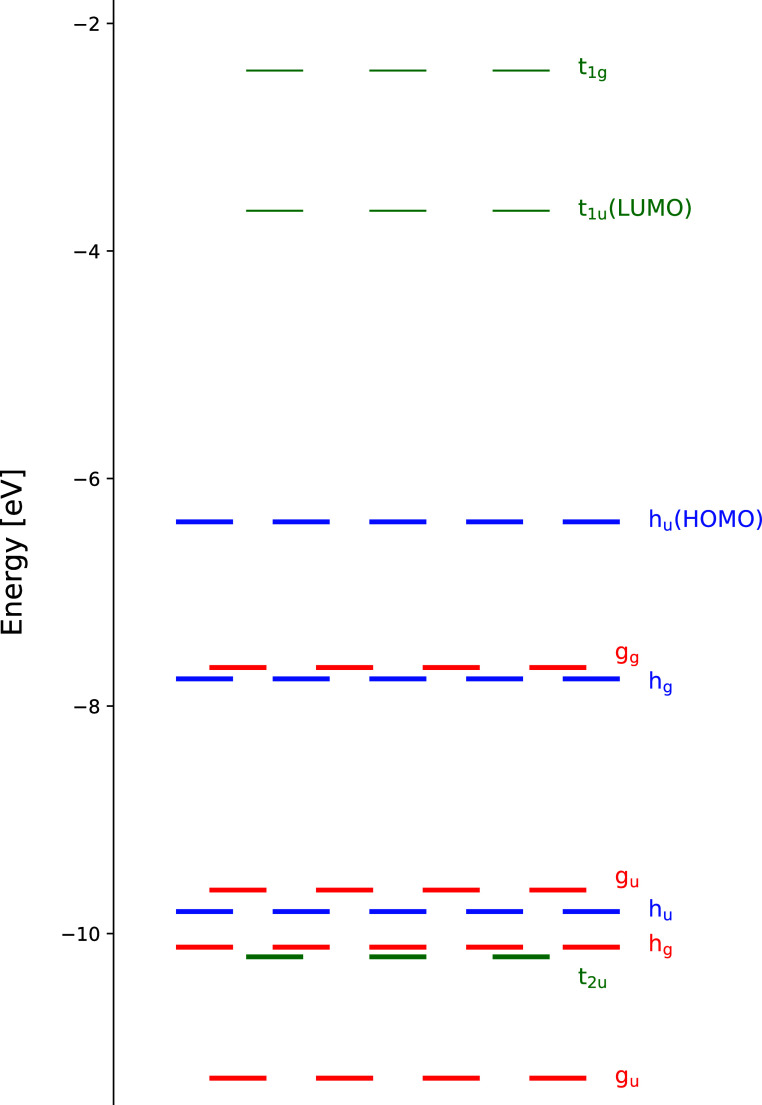
MO energy diagram of the 35 highest occupied (thick bars) and 6 lowest unoccupied (thin bars) MOs of the C_60_ molecule, calculated at the B3LYP/aug-cc-pVDZ level of theory. The irreps of the *I*_h_ symmetry group corresponding to individual orbital groups are also provided.

Therefore, before comparing the HHG spectra obtained using different real-time time-dependent methods, we must first ensure that the results are converged with respect to all three of the above parameters. To achieve this, we conduct a series of benchmark HHG calculations for the C_60_ molecule at both considered carrier frequencies, at the RT-TDTDA-B3LYP level of theory. We test the performance of three basis sets: the minimal STO-3G basis set and the double-ζ Dunning cc-pVDZ basis set and its singly augmented variant, aug-cc-pVDZ. Due to the icosahedral symmetry of C_60_, almost all of its MOs belong to degenerate energy levels, with the HOMO level exhibiting 5-fold degeneracy (Figure 1). We determine the optimal configurational space by employing a full virtual orbital space and gradually expanding the active occupied orbital space, starting from five HOMO orbitals and adding one occupied MO shell at a time. Finally, we perform every calculation using seven different values of the *d* parameter, 10, 50, 100, 150, 200, 250, and 300 bohr, and with the finite lifetime model turned off (which is equivalent to setting *d* → ∞).

**Figure 2:**
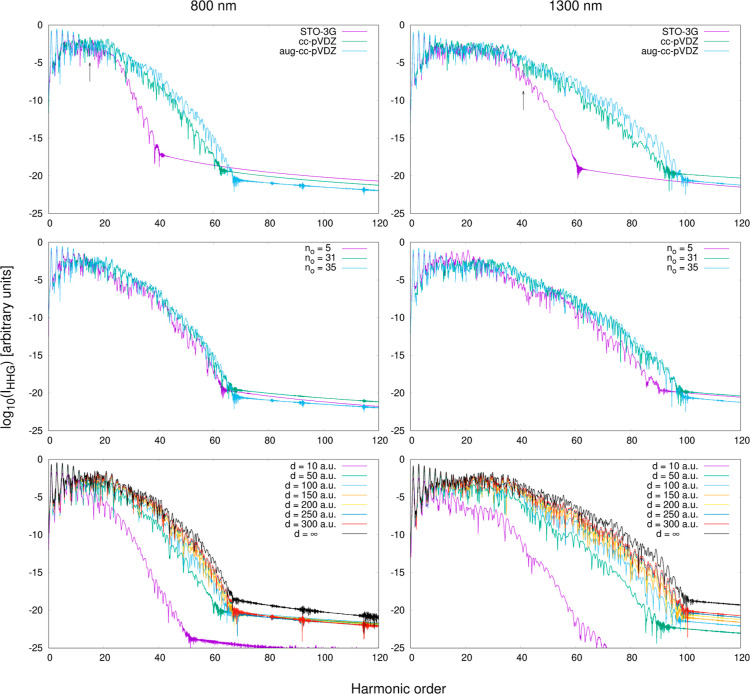
Results of the benchmark calculations of HHG in C_60_ at the RT-TDTDA-B3LYP level of theory. Top row: comparison of the HHG spectra obtained using three different basis sets, with *n*_o_ = 35 and *d* = 200 au. Black vertical arrows denote the positions of the HHG cutoff, as predicted by the 3SM. Middle row: comparison of the HHG spectra obtained in the aug-cc-pVDZ basis set using different numbers of active occupied MOs, with *d* = 200. For better clarity, only the results for *n*_o_ = 5, 31, and 35 are shown. Bottom row: comparison of the HHG spectra obtained in the aug-cc-pVDZ basis set using different values of the *d* parameter, with *n*_o_ = 35.

The results of these preliminary calculations are presented in Figure 2. The two top plots show a comparison of three considered basis sets. It can be seen that the STO-3G basis set provides a significantly different depiction of HHG in comparison with the Dunning basis sets. Specifically, it predicts an abrupt drop in the HHG intensity near the cutoff energy predicted from the three-step model, *E*_cut_ = *I*_*p*_ + 3.17(*E*_0_^2^/4ω_0_^2^), at both wavelengths. In contrast, both cc-pVDZ and aug-cc-pVDZ spectra show a sizable cutoff extension, accompanied by a more gradual reduction in the HHG intensity relative to the harmonic order. This result is more in line with previous theoretical calculations of HHG in fullerenes and with experimental observations. Interestingly, the inclusion of the diffuse basis functions has a noticeably less pronounced effect on the description of HHG compared with the addition of the polarization functions. This is evident as the spectra obtained in the cc-pVDZ and aug-cc-pVDZ basis sets do not differ significantly in terms of their overall shape. The only distinctions are that the latter basis yields slightly higher peak intensities in the high-energy part of the spectrum and is capable of describing several additional peaks. Since these differences are, nonetheless, noticeable, in the further calculations, we use the largest aug-cc-pVDZ basis set.

The two middle plots in Figure 2 depict the influence of the number of active occupied MOs *n*_o_ on the obtained HHG spectra. When increasing the active occupied space, we were able to achieve convergence at approximately *n*_o_ ≈ 30, and as can be seen, at both wavelengths, the spectra obtained with *n*_o_ = 31 and *n*_o_ = 35 are nearly identical to each other. Such an outcome is consistent with the somewhat intuitive reasoning that the π band, consisting of 60 *p*-type orbitals of sp^2^-hybridized carbon atoms, should be most strongly involved in the HHG process because the electrons occupying it can move freely throughout the entire molecule. However, to ensure that the computed spectra are truly converged with respect to *n*_o_, in the subsequent calculations, we use the highest considered number of 35 occupied MOs. In the plots, we also show the results for *n*_o_ = 5, which in our case is the closest to the SAE model. Although not dramatically different from the converged ones, the spectra obtained with *n*_o_ = 5 are of noticeably inferior quality with the decreased HHG intensity and artificial local minima in the high-energy region. While some of the earliest HHG calculations on fullerenes based on the strong-field approximation considered only electronic transitions from the HOMO level,^82,83^ our results indicate that this may not be the optimal strategy.

Finally, in the two bottom plots, we present the dependence between the value of the *d* parameter in the finite lifetime model and the computed HHG signal. It is evident that applying the two lowest *d* values leads to a significant reduction in the HHG intensity and a decrease in the number of peaks, indicating that the absorption model interferes with the HHG process. The calculated HHG response stabilizes at much higher values of *d* ≈ 150 bohr. The spectra obtained with *d* = 150, 200, 250, and 300 bohr exhibit consistent peak shapes and only marginal differences in overall intensity. Therefore, we pick an intermediate value of *d* = 200 bohr for further calculations. It is worth mentioning that the spectra shapes, once converged with respect to *d*, are not identical to the spectra shapes obtained without the finite lifetime model. This implies that the absorber effectively eliminates the wave function reflections arising from the basis set incompleteness as intended, without hindering the HHG efficiency.

At this point, it is important to address the limitations of using Gaussian orbitals in real-time propagations. Previous theoretical works employing quantum chemical methods coupled to Gaussian basis sets to model intense field processes have shown that although standard basis sets usually manage to reproduce lower harmonics, achieving a more comprehensive description of electron dynamics requires the addition of a certain number of highly diffuse and oscillatory functions.^59,60,62,64,65,70,181^ These functions, often tailored for this specific purpose, e.g., by fitting Gaussian functions to Slater orbitals^64,182^ or to Coulomb wave functions,^183,184^ serve a dual role in correcting the laser-driven dynamics. First, by covering the space between the molecular volume and the absorber region, they provide a more accurate description of electron trajectories, particularly when the electron travels significant distances from the molecular center. Second, as HHG involves rapid sequences of electronic transitions and absorption of multiple photons, augmenting the basis set to include functions with higher angular quantum numbers helps capture processes with large changes in the total angular momentum. Besides appending diffuse orbitals to the existing atomic centers, another effective strategy for enhancing the basis set completeness involves adding more functions in the form of ghost atoms.^60,71,75^

It should be emphasized, however, that the aforementioned studies focused on simulating electron dynamics in small systems where HHG is confirmed to occur, at least to some extent, in accordance with the three-step model. On the other hand, in our case, even the addition of the standard Dunning diffuse functions has very little effect on the obtained HHG spectra. We can therefore infer that in contrast to atoms and small molecules, HHG in C_60_ mainly occurs on the surface of the fullerene, so adding more diffuse functions is not necessary. This is further evidenced by the high optimal *d* value that greatly exceeds the maximum electron excursion distance under the considered laser conditions (equal to 11.6 bohr at 800 nm and 30.7 bohr at 1300 nm), even when extended by the largest internuclear distance in C_60_ (≈13.4 bohr). The orbital ionization rate in the finite lifetime model, , is interpreted as an inverse of the time required for an electron with kinetic energy ϵ_*a*_ to travel a distance *d*. In this context, the parameter *d* can be considered equivalent to the distance between the molecule and the starting point of the complex absorbing potential. However, from Figure 2, it is evident that setting *d* to be similar to the electron excursion distance results in an overestimation of absorption. This overestimation occurs due to the assignment of excessively short lifetimes to virtual MOs, akin to placing the complex absorbing potential too close to the molecule. This effect implies that in C_60_, the proportionality between the virtual MO energy and the distance that an electron occupying this MO can travel within a given amount of time is no longer valid. The probable cause is that even virtual MOs with relatively high energies are still localized in the vicinity of the molecular surface. This indirectly confirms that fullerene HHG results from oscillations of the electron density within the molecule and cannot be fully described by the three-step model.

Lastly, when dealing with such a large system, expanding the basis set to achieve convergence of results is hindered by technical limitations. The rank of the matrices **A** and **B**, constructed in the aug-cc-pVDZ basis set using the full virtual space and active occupied space with *n*_o_ = 35, is equal to 42,000. Full diagonalization of larger matrices, although technically feasible, would demand considerable time and resources. To the best of our knowledge, this is already the highest number of excited states reported for calculations employing real-time time-dependent wave function methods. Moreover, in the case of the C_60_ fullerene, the aug-cc-pVDZ basis set is already on the verge of linear dependencies.

### 3.2 Comparison of Different Exchange–Correlation Potentials

In this section, we compare the HHG spectra computed using the B3LYP functional with those obtained using the CAM-B3LYP functional and calculated at the HF level. At this point, we adhere to the CIS/TDA approximation. Let us briefly remind the reader that while B3LYP and CAM-B3LYP share the same correlation functional, they differ in terms of the exchange functional. B3LYP contains a fixed portion of 20% HF exchange, which is known to lead to incorrect behavior of this functional in the long range. Therefore, CAM-B3LYP has been proposed as a range-separated version of B3LYP, in which the percentage of HF exchange depends on interelectronic distance and varies from 19% at the short range to 65% at the long range.^173^ While still not making it asymptotically correct, this considerably improves the long-range behavior of the functional.

**Figure 3:**
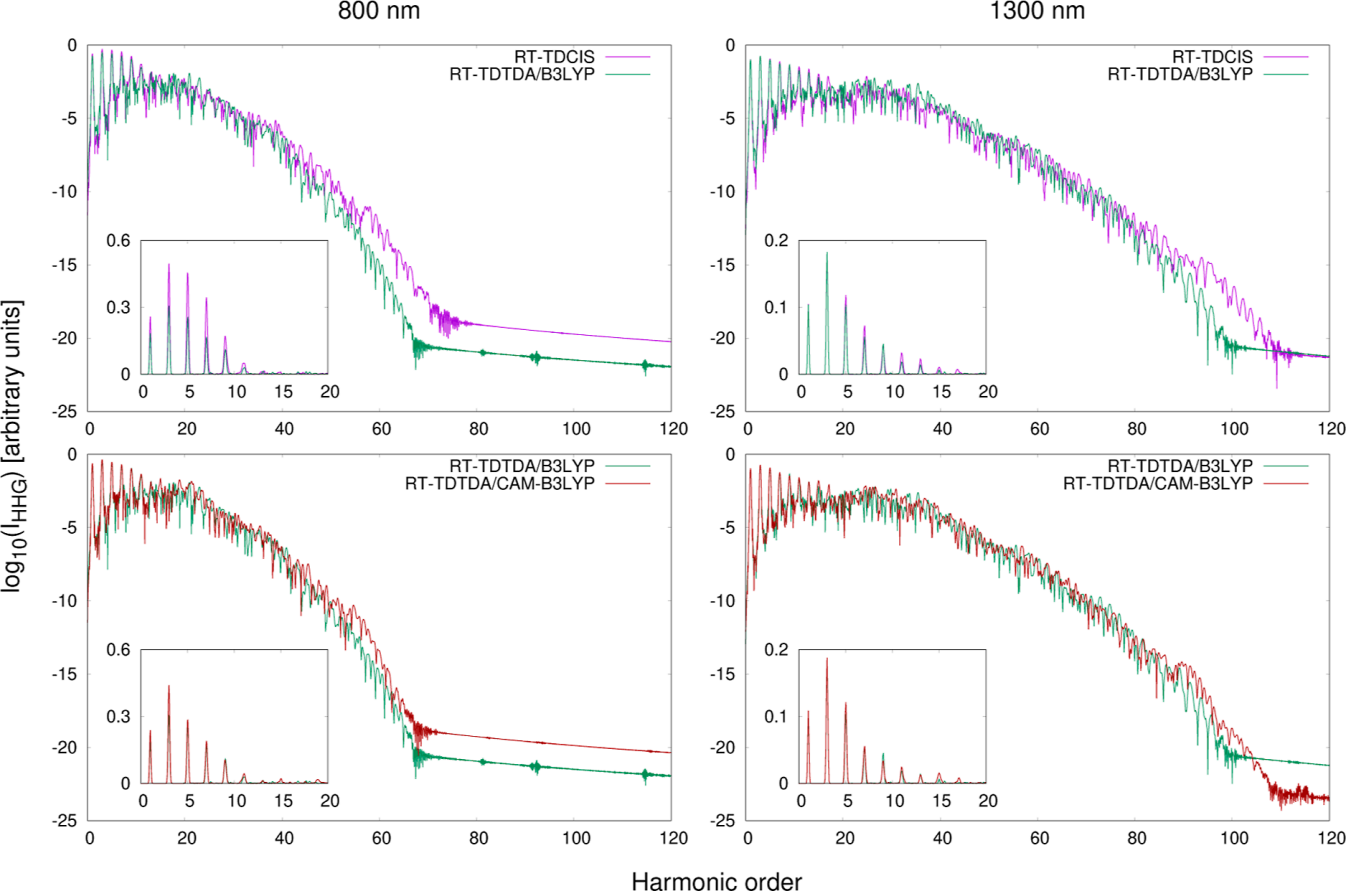
Comparison of the HHG spectra of C_60_ calculated in the aug-cc-pVDZ basis set at the RT-TDCIS, RT-TDTDA-B3LYP, and RT-TDTDA-CAM-B3LYP levels of theory. The insets show the lowest-energy regions of the spectra, plotted in the linear scale.

The HHG spectra obtained from RT-TDCIS, RT-TDTDA-B3LYP, and RT-TDTDA-CAM-B3LYP are shown in Figure 3. All three methods provide spectra of comparable quality in terms of overall shape and intensity. However, we can distinguish two regions where certain systematic differences can be observed depending on the *f*_*xc*_ used. The first of them is the lowest-energy part of the spectrum, where individual peaks are most clearly visible. It can be seen that RT-TDCIS predicts significantly higher intensities of peaks up to the 20th harmonic order compared to both variants of RT-TDTDA, especially at 800 nm. In our opinion, this part of the spectrum is primarily influenced by the short-range interactions, specifically the short-range correlation. The peaks with the lowest energy levels represent transitions to and from the least energetic excited states, during which the excited electron remains in close proximity to the molecule. Therefore, the dynamical correlation effects are expected to be most prominent in this region. Since the correlation part of B3LYP is the same as that of CAM-B3LYP, the peak intensities in this part of the spectrum predicted by these two functionals are much more similar to each other. We have already discussed the effect of decreased HHG intensity due to correlation effects in our works on smaller systems.^65,70^

**Figure 4:**
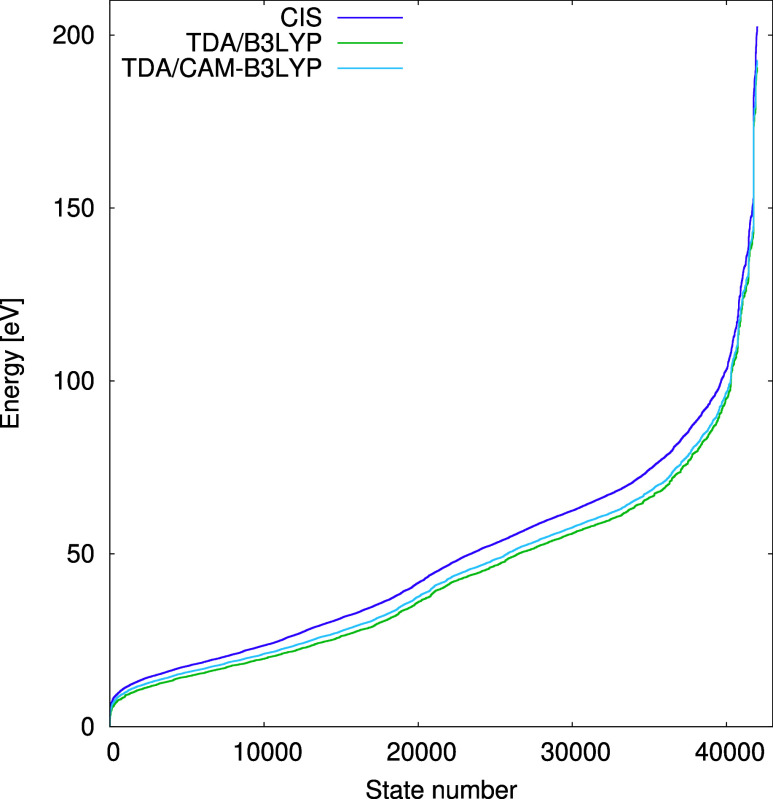
Distributions of the excitation energies obtained using CIS, TDA-B3LYP, and TDA-CAM-B3LYP in the aug-cc-pVDZ basis set.

The second region encompasses the highest-energy part of the spectrum, extending beyond the 40th harmonic at 800 nm and beyond the 80th harmonic at 1300 nm. By analogy, we suspect that the description of this region is mainly governed by long-range interactions. It can be noticed that the intensity of the spectrum “tail” is more or less proportional to the amount of HF exchange in the exchange–correlation functional. RT-TDCIS, which provides an asymptotically correct description of exchange effects, predicts the most intense peaks in this region. In contrast, RT-TDTDA-B3LYP, which is the least accurate at the long-range limit, predicts peaks of the lowest intensity. Additionally, at 1300 nm, both RT-TDCIS and RT-TDTDA-CAM-B3LYP can describe a few more peaks than RT-TDTDA-B3LYP. A similar pattern can also be seen when comparing the distributions of excited states obtained using different exchange–correlation potentials (Figure 4). Introducing correlation by replacing the HF potential with B3LYP lowers nearly all excitation energies, but the correction of the long-range exchange has a counteracting effect, causing a slight shift back toward higher values.

### 3.3 Influence of Deexcitation Effects on the HHG Response

**Figure 5:**
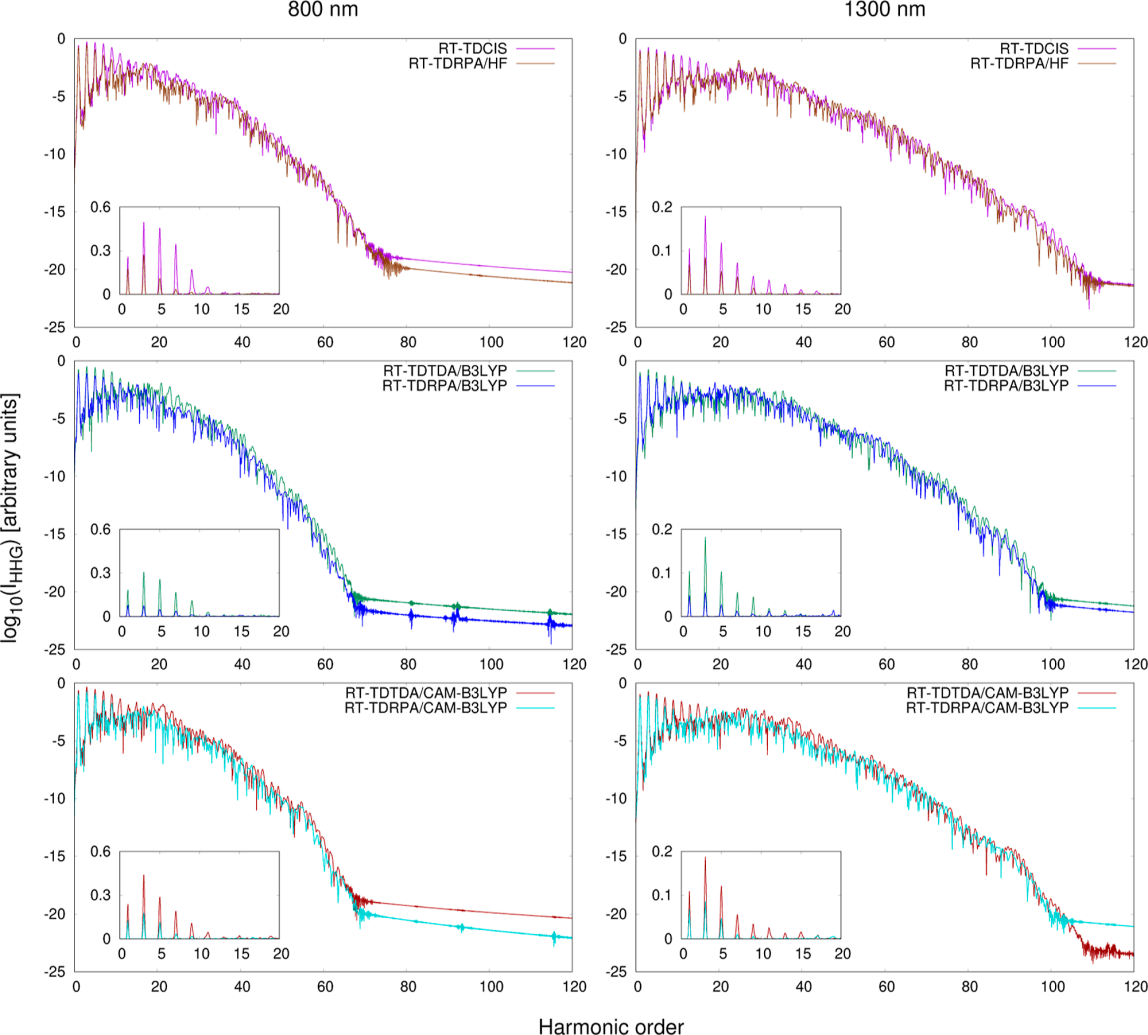
Comparison of the HHG spectra of C_60_ calculated in the aug-cc-pVDZ basis set: RT-TDCIS vs RT-TDRPA-HF (top row), RT-TDTDA-B3LYP vs RT-TDRPA-B3LYP (middle row), and RT-TDTDA-CAM-B3LYP vs RT-TDRPA-CAM-B3LYP (bottom row). The insets show the lowest-energy regions of the spectra, plotted in the linear scale.

In Figure 5, we compare the HHG spectra obtained in the TDA/CIS approximation with those obtained using the full RPA framework separately for each of the three considered correlation–exchange potentials. Including the **B** matrix when solving the linear-response problem has a much smaller impact on the description of HHG than the choice of the exchange–correlation potential does. In all cases, the spectra computed using RPA and TDA/CIS eigenstates nearly overlap with each other. The only systematic effect is that RT-TDRPA predicts a slightly lower HHG intensity across the entire spectrum, which is more visible at 800 nm. Also, this effect is subtly more pronounced for RT-TDRPA-DFT than for RT-TDRPA-HF. The only exception can be observed for CAM-B3LYP, where the background level of the RT-TDRPA spectrum is upshifted compared to that of the RT-TDTDA spectrum, resulting in a reduction of the number of described peaks in the highest-energy region.

**Figure 6:**
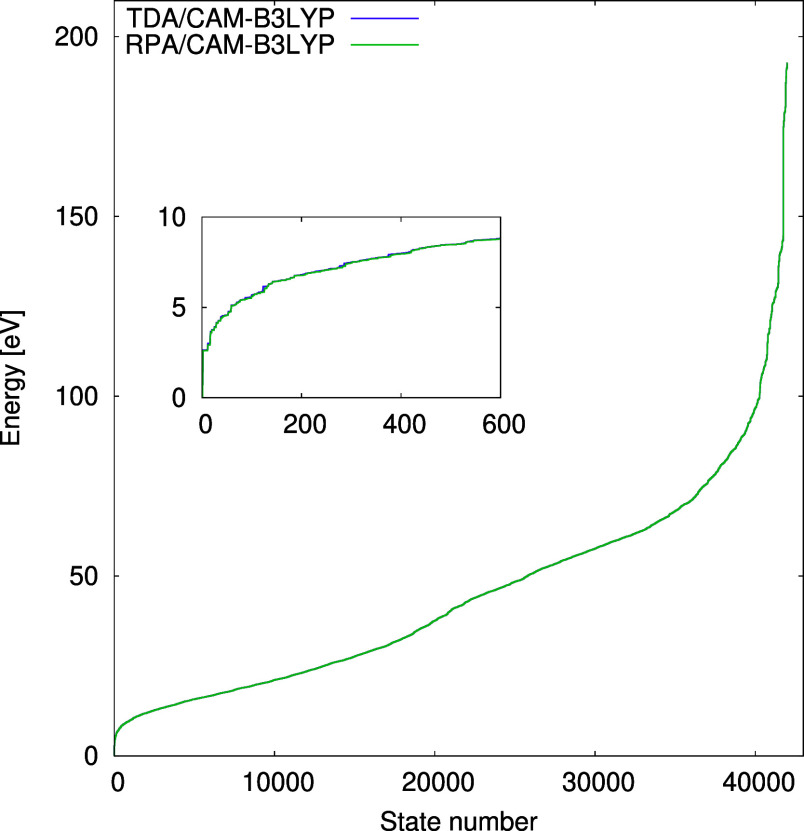
Distributions of the excitation energies obtained using TDA-CAM-B3LYP and RPA-CAM-B3LYP in the aug-cc-pVDZ basis set. The inset shows the zoom of the 600 lowest excitation energies.

Interestingly, RT-TDRPA most significantly reduces the intensity of HHG in the lowest-energy region of the spectra, where the intensities of the first few peaks are 2–4 times lower compared to those of RT-TDTDA and RT-TDCIS. Although we explained the similar differences between RT-TDTDA and RT-TDCIS spectra by the correlation effects, such an explanation may not be appropriate in this case. While RPA is recognized to be a correlated method for the ground state, it is a matter of debate as to whether this property applies to excited states, as well. Recently, Berkelbach has shown that LR-TDHF is equivalent to a variant of EOM-CCD, in which the CCD ground state is taken as a reference state, but only single excitations are considered when solving the equations of motion.^185^ This implies that the RPA excitation energies do not include any additional correlation effects beyond those already accounted for in the exchange–correlation kernel, especially given that in all flavors of RT-TDRPA, we use a single-reference ground state. We reach a similar conclusion when analyzing the excited state distributions obtained from RPA and TDA/CIS calculations, which are almost identical to each other (Figure 6). We believe that the differences between the peak intensities may instead come from the differences in the dipole moments and dipole transition moments between TDA/CIS and RPA states. It is known that LR-TDHF and LR-TDDFT obey the Thomas–Reiche–Kuhn sum rule, unlike CIS and TDA.^150^ Therefore, one should anticipate an improved description of the ground-to-excited transitions in the former two methods. On the other hand, we calculate the excited-to-excited transition moments between LR-TDHF and LR-TDDFT using the approximate formula 15, whereas the analogous eq 17 is a valid expression for CIS and TDA eigenstates. Due to these two potential sources of errors, it is not possible to definitively determine which approach, with or without including the **B** matrix, allows for a better description of electron dynamics.

**Figure 7:**
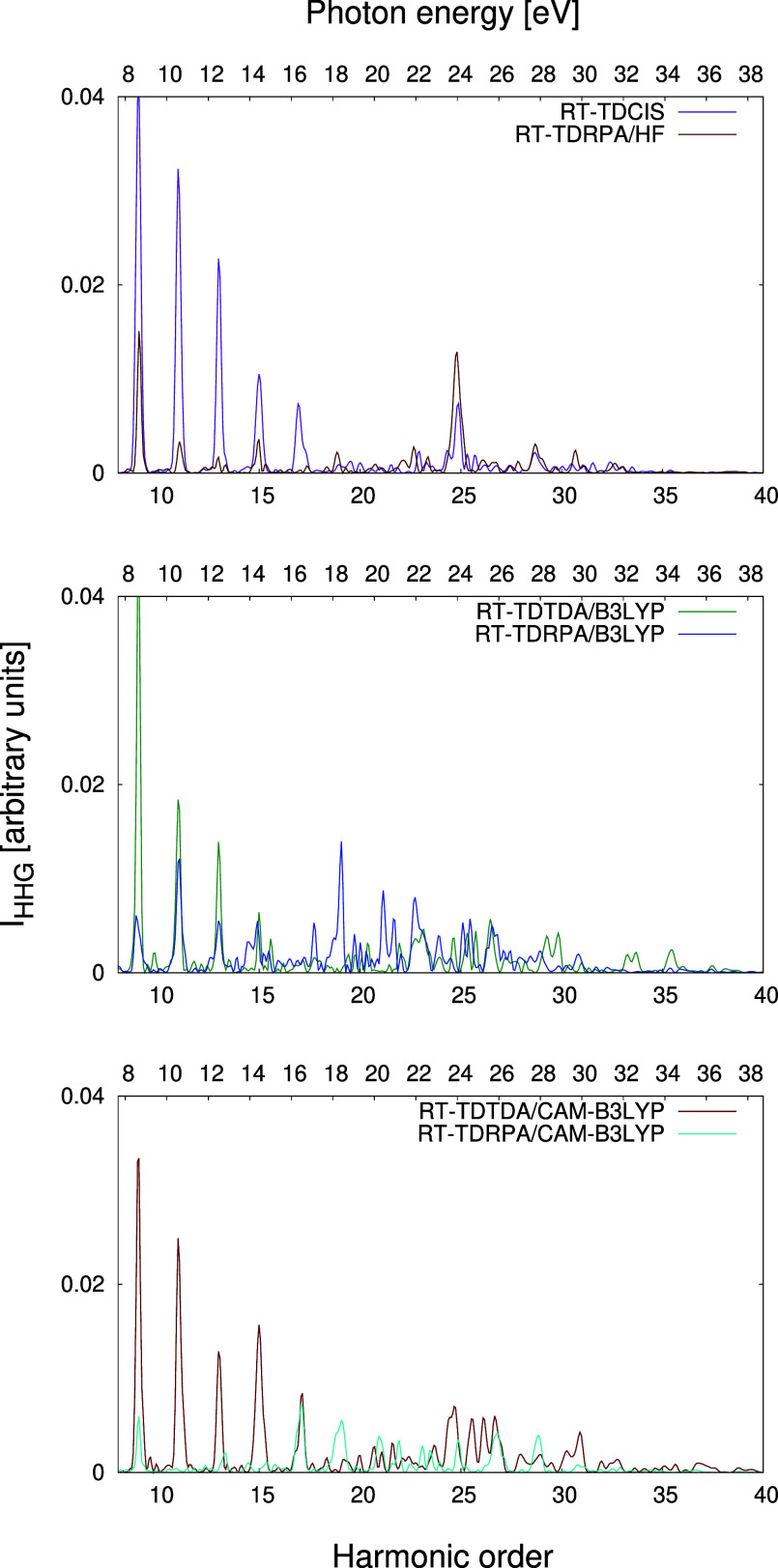
Excerpts of the spectra computed at 1300 nm from Figure 5, covering the region in which the GDR is expected to occur.

When analyzing the computed HHG signals, we noticed that some of the spectra calculated at 1300 nm display a local maximum of intensity between the 15th and 30th harmonic orders. At this wavelength, this region corresponds to photon energies of 14–29 eV. This is the energy range in which the giant dipole resonance (GDR) can be observed in the photoionization spectra of C_60_.^186−190^ GDR can be explained as a shift from individual single-electron excitations to a collective electronic oscillation at the fullerene surface, occurring at a specific exciting wavelength. This is accompanied by the opening of additional ionization channels, alternative to single-electron channels.^187^ Since HHG is closely tied to photoionization, GDR also manifests as an amplification of the harmonic radiation at the corresponding energy.^81^ This phenomenon has been previously reproduced theoretically in both HHG and photoionization spectra using simple RT-TDDFT and numerical models.^88,191,192^ On Figure 7, we take a closer look at the region of the spectra in which GDR is expected to be observed. Surprisingly, it is most clearly visible in the RT-TDCIS and RT-TDRPA-HF spectra, where a single peak at the 25th harmonic order (corresponding to approximately 23.8 eV) is significantly enhanced. RT-TDTDA-CAM-B3LYP provides a not too dissimilar picture, as an enhancement of a group of peaks between the 24th and 27th harmonic orders (22.9–25.7 eV) can be observed. Both of these results are in reasonable agreement with experimental works that predict the GDR maximum at 20–22 eV for C_60_ and 21–24 eV for C_60_^+^.^187,188,190^ RT-TDRPA-B3LYP predicts the strongest enhancement of the 19th peak, corresponding to a somewhat lower energy of 18.1 eV. Finally, practically no enhancement can be seen in the RT-TDTDA-B3LYP and RT-TDRPA-CAM-B3LYP spectra. This simple test allows us to draw the conclusion that the full RPA treatment of the excited states may indeed result in a less accurate description of the electron dynamics in C_60_, at least when a Kohn–Sham determinant is employed as a reference, as evidenced by the case of the CAM-B3LYP calculations. Furthermore, the fact that B3LYP is unable to predict the GDR at the RT-TDTDA level and provides its incorrect position at the RT-TDRPA level may indicate that long-range exchange effects are particularly important for describing the global resonances in C_60_. Unfortunately, we are unable to detect the GDR-related enhancement at 800 nm as, at this wavelength, it should be located at lower harmonic orders, where the overall HHG intensity is several orders of magnitude higher.

### 3.4 Semiempirical HHG Calculations

**Figure 8:**
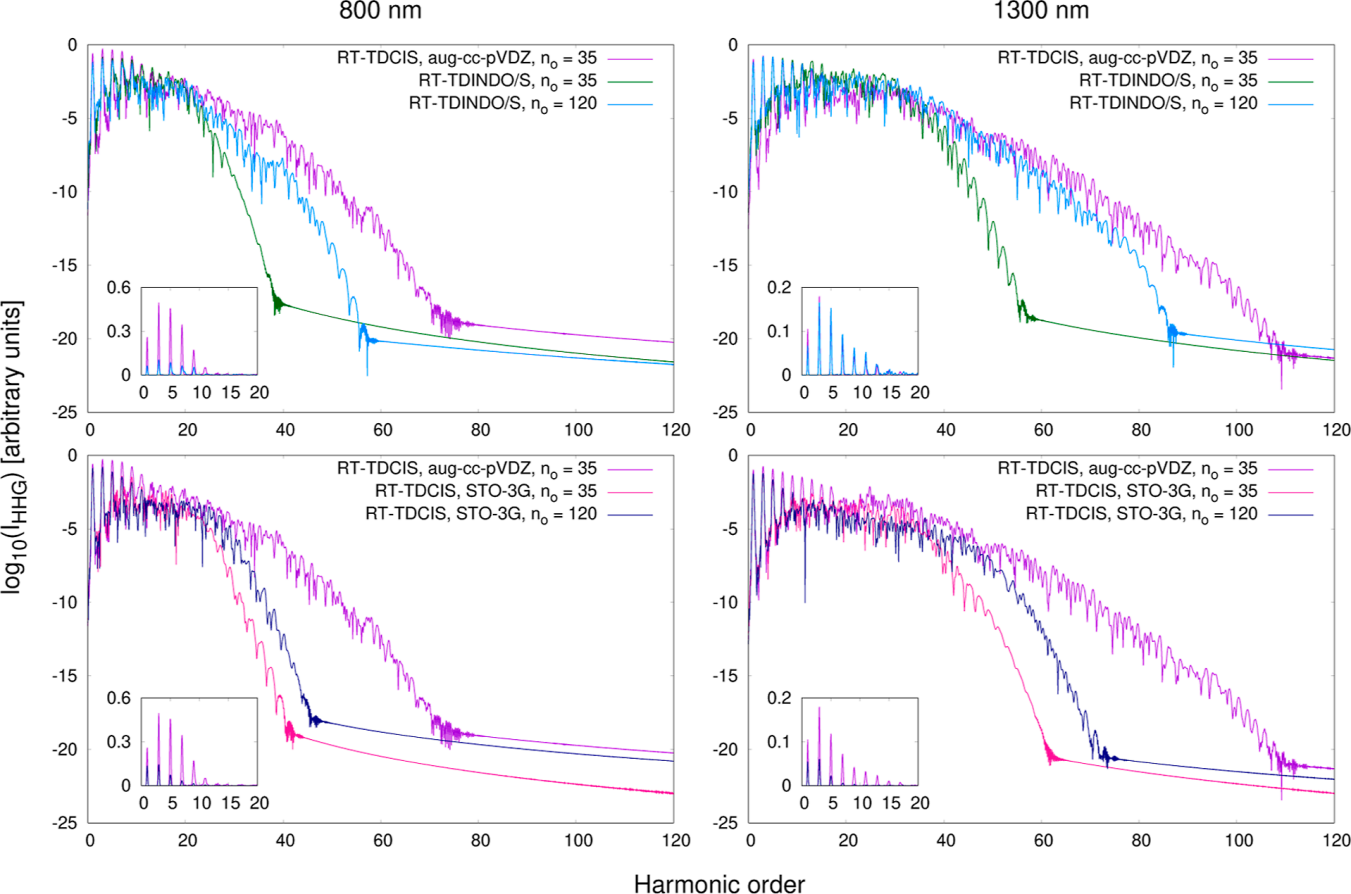
Top row: comparison of the HHG spectra computed using RT-TDCIS in the aug-cc-pVDZ basis set and using RT-TDINDO/S within two different active occupied spaces. Bottom row: comparison of the HHG spectra computed using RT-TDCIS in the aug-cc-pVDZ basis set and in the STO-3G minimal basis set within two different active occupied spaces.

Finally, in this section, we analyze the results obtained from the RT-TDINDO/S propagations. As mentioned earlier, in RT-TDINDO/S simulations, we employ the Slater minimal basis set compatible with semiempirical methods, while the size of the active occupied space and the value of the *d* parameter remains the same as in the ab initio and DFT-based calculations. Since INDO/S can be considered a semiempirical counterpart of CIS, a natural choice is to compare the computed spectra with the RT-TDCIS ones. Such a comparison is shown in two upper plots in Figure 8, where the purple curves represent RT-TDCIS results in the aug-cc-pVDZ basis set (henceforth referred to as the reference results) and the green curves represent RT-TDINDO/S results. RT-TDINDO/S successfully reproduces most of the peaks in the low-energy portion of the spectra. This is a commendable result considering the approximate nature of the INDO/S Hamiltonian. Nonetheless, the picture it provides at higher energies closely resembles that of RT-TDTDA-B3LYP in the minimal basis set (shown in Figure 2), with a sudden decrease in the HHG intensity at too low energies. At first glance, this may suggest that RT-TDINDO/S is not suitable for HHG modeling. However, when performing calculations, we observed that unlike the reference results, the RT-TDINDO/S spectra are not converged with respect to the number of active occupied MOs at *n*_o_ = 35, and further expanding the active occupied space leads to a gradual improvement in their quality. Therefore, we also perform a second series of calculations, this time allowing excitations from all 120 occupied INDO/S orbitals (light blue curves in Figure 8). This significantly improves the HHG depiction in the high-energy range. The positions of the last described peaks are upshifted from the 37th to the 57th harmonic order at 800 nm and from the 57th to the 87th harmonic order at 1300 nm. Additionally, the overall intensity of HHG now aligns more closely with that predicted by RT-TDCIS over a much broader energy range. Interestingly, RT-TDINO/S performs notably better at 1300 nm, where a good agreement with the RT-TDCIS curve is observed up to approximately the 50th harmonic order and the intensities of the lowest-energy peaks are very closely reproduced. At 800 nm, RT-TDINDO/S predicts two regions where the intensity of HHG significantly decreases, the first one around the 23rd harmonic order and the second around the 43rd harmonic order, leading to larger discrepancies with the RT-TDCIS predictions.

The above observations prompt a question about whether the observed improvement of RT-TDINDO/S performance is genuinely attributable to the effectiveness of the semiempirical Hamiltonian or if it is merely a consequence of expanding the configurational space. To address it, we conduct analogous calculations at the RT-TDCIS level, using the STO-3G basis set and the same two active occupied spaces. The STO-3G basis set serves as our closest analogue to the INDO/S Slater minimal basis set as both provide the same number of virtual MOs. Thus, for a given *n*_o_, they yield exactly the same number of excited states, totaling 4200 for *n*_o_ = 35 and 14,400 for *n*_o_ = 120. The results are presented in the two bottom plots of Figure 8. While switching to a larger *n*_o_ modestly extends the cutoff at both intensities, the magnitude of this extension is nowhere near what is observed in the RT-TDINDO/S spectra. The position of the last described peak is shifted upward by about 5 harmonic orders at 800 nm and by about 12 harmonic orders at 1300 nm.

**Figure 9:**
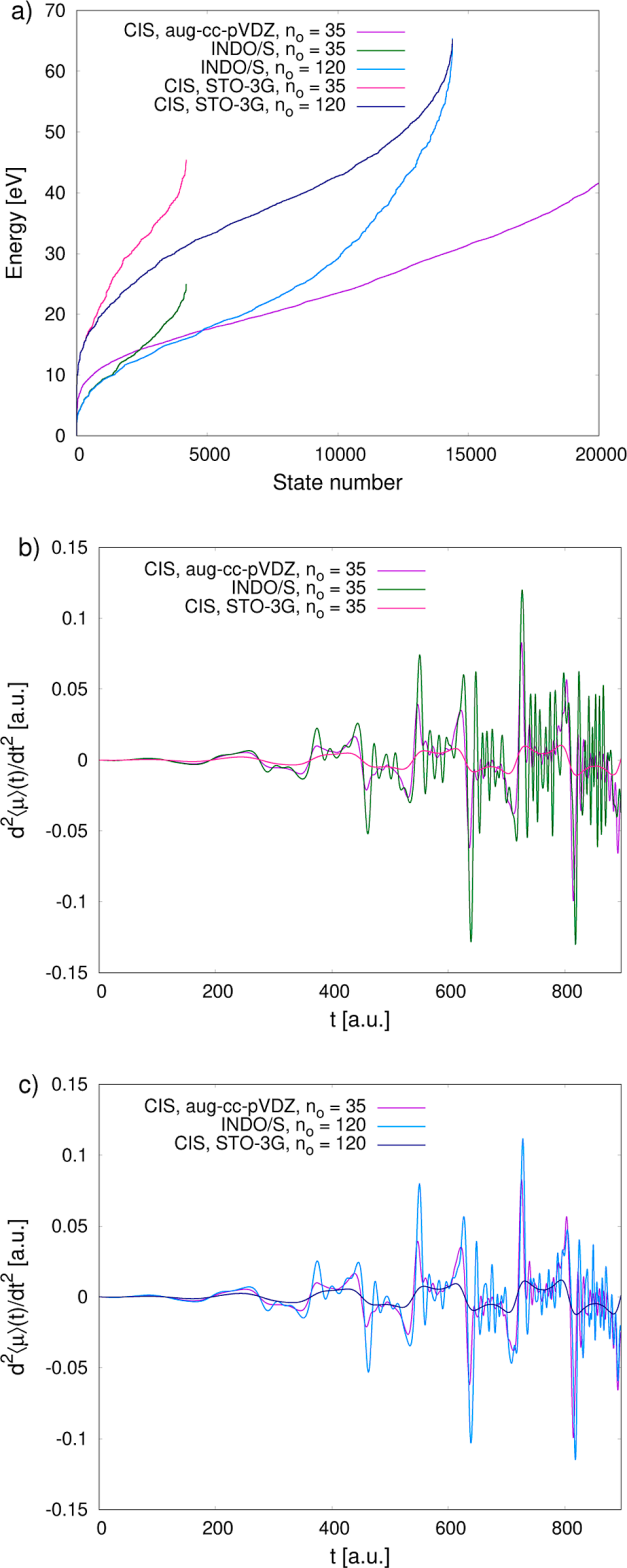
(a) Distributions of excitation energies calculated with CIS in the aug-cc-pVDZ and STO-3G basis sets and with INDO/S. (b), (c) Time-resolved dipole acceleration during the first five optical cycles of the applied laser pulse of 1300 nm, obtained from the RT-TDCIS/aug-cc-pVDZ, RT-TDCIS/STO-3G, and RT-TDINDO/S propagations. In the two latter approaches the active occupied space contains either 35 (b) or 120 (c) highest occupied MOs.

To better understand the differences between the pictures provided by RT-TDCIS/STO-3G and RT-TDINDO/S, let us analyze the distributions of excited states obtained at these two approaches as well as the trajectories of the dipole acceleration over time, presented in Figure 9. At both *n*_o_ = 35 and *n*_o_ = 120, the excitation energies obtained with TDINDO/S and with CIS in the Dunning basis set lay in a similar energy range, as opposed to excitation energies from CIS in the STO-3G basis set, which are severely overestimated (Figure 9a). This has a direct effect on the time-resolved dipole acceleration. The excited states of CIS/aug-cc-pVDZ and RT-TDINDO/S are similarly accessible to the electrons, resulting in a roughly similar magnitude of the optical response (Figure 9b). However, the dipole acceleration from RT-TDINDO/S with *n*_o_ = 35 also exhibits a noticeable level of numerical noise, which indicates that the excited space is too confined and the wave function undergoes unphysical reflections. Increasing *n*_o_ augments the pool of available electronic transitions and enhances the overall flexibility of the excited space, aligning a larger portion of the INDO/S eigenspectrum with the reference one (Figure 9a). This significantly reduces the noise, leading to a much better agreement in terms of the optical response (Figure 9c). The oscillations in the dipole acceleration trajectory from RT-TDINDO/S with *n*_o_ = 120 are still slightly overestimated, but this is probably due to the fact that the excitation energies of the first few thousand excited states are marginally lower than that in CIS/aug-cc-pVDZ, resulting in their slight overpopulation.

On the other hand, the eigenspectrum of CIS/STO-3G with *n*_o_ = 35 is not only overly constrained but also barely any excited states are energetically available in the laser field. This leads to a smoother, less oscillatory dipole acceleration curve with a much smaller overall magnitude, which explains why the intensities of the first few harmonics in the RT-TDCIS/STO3G spectra are lower than those in both the RT-TDINDO/S and reference results. Naturally, expanding the active occupied space in this case also increases the number of  eigenstates. However, as the energy range of these new states remains largely unaltered, a majority of them still remain inaccessible to the electrons. Therefore, transitioning from *n*_o_ = 35 to *n*_o_ = 120 yields practically no improvement in the time-resolved dipole acceleration.

We presume that the lack of a sufficiently high number of energetically available electronic states—caused by a too small excited space dimension in the case of RT-TDINDO/S with *n*_o_ = 35, the overestimation of excitation energies in the case of RT-TDCIS/STO-3G with *n*_o_ = 120, and both of these factors in the case of RT-TDCIS/STO-3G with *n*_o_ = 35—is also the reason why all three levels of theory fail to describe HHG in the high-energy range. This of course cannot be directly inferred from the dipole acceleration plots as the highest harmonics are characterized by far too high frequencies and extremely low intensities. However, a comparison of the optical responses yielded by the different levels of theory offers a valuable overview of the excited space quality.

**Figure 10:**
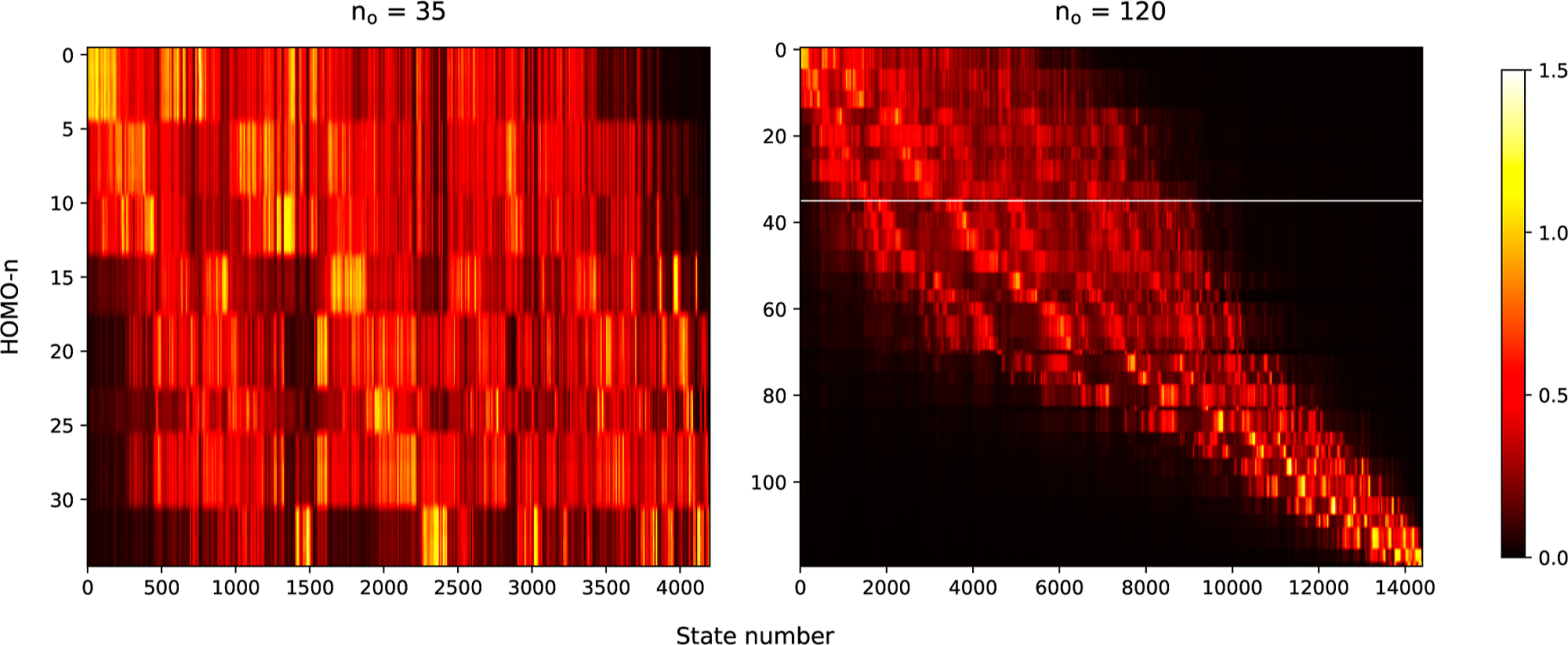
Graphical representation of the contributions from excitations from different occupied orbitals to different INDO/S excited states, in the active occupied spaces with *n*_o_ = 35 (left) and *n*_o_ = 120 (right). An entry corresponding to the *i*-th occupied MO and the *k*-th excited state is calculated as . The white horizontal line on the right plot denotes the HOMO-35 orbital for an easier comparison with the left plot.

It may be debatable as to whether it is justified to include the excitations from inner orbitals in the RT-TDINDO/S simulations, especially since we have previously shown that including them is not required for the convergence of the HHG spectra in a larger basis set. However, an examination of the excited configurations’ contributions to particular excited states (Figure 10) reveals that augmenting *n*_o_, while enabling transitions from inner MOs, also leads to a notable increase in the number of states with significant involvement of outer-shell excitations, raising their count to over 8000. At the same time, only the excited states above roughly the 8000th eigenspectra exhibit notable deviations from the reference eigenspectrum in Figure 9a. This leads to the conclusion that approximately doubling the subspace of valence-shell excited states is most likely responsible for the improvement in the description of HHG in the high-energy region. It is also important to highlight that semiempirical methods usually yield wave function structures that differ somewhat from those obtained with ab initio approaches. Nonetheless, they still offer reasonable values of observables, thanks to the effective parametrization of the operators’ matrix elements that allows for overcoming the limitations of the minimal basis set. This property is routinely utilized in the time-independent semiempirical calculations.^158^ Since INDO/S has been optimized to provide approximate excitation energies and dipole matrix elements, which are the only components essential for the real-time propagation, it is reasonable to infer that this trait applies to the nonperturbative regime as well.

While RT-TDINDO/S may not perfectly replicate the HHG spectra obtained in a larger basis set, it has a significant advantage shared with its time-independent counterpart, which is low computational requirements. For instance, on a 24-core Intel Xeon Platinum 8268 CPU, the average timings for the RT-TDCIS/aug-cc-pVDZ simulations at 1300 nm were 1908 min for diagonalizing the **H**_**0**_ and **μ** matrices and 533 min for the real-time propagation. In contrast, for the RT-TDINDO/S simulations with an expanded active occupied space, the corresponding timings were only 87 and 97 min, respectively. The same timings are achieved for the RT-TDCIS/STO-3G simulations as the order of the matrix eq 18 is equal for both methods. Thus, RT-TDINDO/S offers a computational cost comparable to that of the ab initio methods in a minimal basis set while simultaneously providing a quality of results much closer to that of more sophisticated levels of theory.

## 4 Conclusions

In this study, we conducted and analyzed a series of calculations of the HHG spectra of the C_60_ fullerene in the nonperturbational regime, employing various quantum chemical methods. These include the real-time time-dependent counterparts of CIS, TDA, and RPA based on either the Hartree–Fock or Kohn–Sham determinant as well as the semiempirical INDO/S method.

The main conclusion that can be drawn from this work is that ab initio and DFT-based methods coupled to Gaussian bases can be successfully applied to model strong-field processes in nanoscale systems. Naturally, these calculations are much more resource-intensive compared to those on atoms and smaller molecules as most of the performed propagations involved expanding the time-dependent wave function in several tens of thousands of states. Nevertheless, owing to the linearity of the real-time propagation equations, the computational cost remains reasonable and represents a modest price for an effectively all-electron picture of the laser-driven dynamics. Our calculations on C_60_ correctly predict that the HHG in fullerenes primarily arises from the oscillations of the electronic density at the molecular surface, with a predominant contribution from the 60 π electrons to this process. We also demonstrated that real-time time-dependent single excitation-based methods can detect more subtle features of the attosecond processes, such as the GDR.

Our results indicate that the quantum chemical description of the electron dynamics primarily depends on the chosen basis set and the active orbital space employed. The influence of the exchange–correlation potential is relatively minor. Nevertheless, we were able to identify some features of the spectra that can be attributed to both correlation and exchange effects. Based on our findings, we can recommend the use of range-separated DFT functionals in the calculations involving systems with a significant role in dynamical correlation. In our case, CAM-B3LYP successfully combines the good approximation to the short-range correlation of B3LYP with the proper treatment of the long-range exchange characteristic with HF-based methods. On the other hand, a full RPA description of the excited electronic states provides practically no improvement over CIS, and may, in fact, lead to inferior results compared to those of TDA based on the Kohn–Sham reference.

Finally, we have demonstrated that the semiempirical INDO/S Hamiltonian, traditionally recognized for offering reasonably accurate approximations to excitation energies, can also be successfully used for modeling strong-field processes in large systems. RT-TDINDO/S outperforms all-electron methods with the same number of employed excited states, yielding results closer in quality to those achievable with larger basis sets, particularly at lower harmonic orders and for relatively long laser wavelengths. This improvement can be attributed to the interplay between the effective parametrization of the semiempirical Hamiltonian and the appropriate choice of the configurational space, which may require the inclusion of additional types of single excitations. These findings position RT-TDINDO/S as a potentially valuable simulation framework for studying even larger systems, which are gaining increasing interest in attosecond physics but exceed the capabilities of the ab initio and DFT-based methods. The applicability of RT-TDINDO/S will be further explored in future works.
